# Visual outcomes of macula-involving rhegmatogenous retinal detachment in patients with and without age-related macular degeneration

**DOI:** 10.1186/s12886-022-02718-y

**Published:** 2022-12-06

**Authors:** P. Barrett Paulk, Dala Eloubeidi, Mark Johnson, Thomas Swain, John O. Mason, Christine A. Curcio, Jason N. Crosson

**Affiliations:** 1 Department of Ophthalmology and Visual Sciences, University of Alabama at Birmingham School of Medicine, 1720 University Boulevard, Birmingham, AL 35223 USA; 2 University of South Alabama College of Medicine, 5795 USA Drive North, CSAB 170, Mobile, AL 36688 USA; 3 Retina Consultants of Alabama, 700 18th Street South, Birmingham, AL 35223 USA

**Keywords:** Macula-off rhegmatogenous retinal detachment, Age-related macular degeneration, Visual acuity

## Abstract

**Background:**

The prognosis for patients with macula-off rhegmatogenous retinal detachment (RRD) and concomitant age-related macular degeneration (AMD) is not well known. The purpose of this study is to compare visual outcomes in macula-off RRD in eyes with AMD versus a group of comparison eyes without AMD.

**Methods:**

This was a retrospective chart review of 1149 patients. A total of 191 eyes met study criteria, 162 non-AMD eyes (controls), and 29 AMD eyes. The main outcome measure was postoperative visual acuity following pars plana vitrectomy (PPV), scleral buckle (SB), or combined PPV/SB in control eyes versus AMD eyes. This was compared using Fisher’s exact test.

**Results:**

There was a statistically significant difference in postoperative visual acuity by AMD status, with those without AMD having a worse visual outcome overall (*p* = 0.0048). A similar percentage of AMD versus non-AMD eyes achieved vision better than 20/40. More patients in the non-AMD group achieved a final visual acuity between 20/40 and 20/200. Of patients with AMD, more had vision worse than 20/200 though 58% maintained functional vision (better than 20/200). Those without AMD had a higher frequency of Count Fingers (CF), Hand Motion (HM), Light Perception (LP), or No Light Perception (NLP) vision (*p* = 0.023).

**Conclusions:**

Though postoperative visual acuity was worse overall in the non-AMD group with a higher frequency of patients having final vision of CF, HM, LP, or NLP, this is likely a function of the difference in sample size and composition between the two groups. Importantly, this study suggests AMD patients can expect similar outcomes to non-AMD patients after RRD repair. We conclude that AMD patients can achieve functional vision after RRD surgery, similar to those without AMD.

**Supplementary Information:**

The online version contains supplementary material available at 10.1186/s12886-022-02718-y.

## Background

Rhegmatogenous retinal detachment (RRD) can lead to devastating visual loss. RRD affects a significant proportion of patients around the world, with an incidence varying between 6.3 to 17.9 per 100,000 [1]. RRD typically occurs at the time of posterior vitreous separation from the retinal surface when vitreous traction causes a tear in the retina. Fluid in the vitreous cavity can then track under the retina and separate the neurosensory retina from the underlying retinal pigment epithelium (RPE), resulting in visual morbidity that can be permanent if not addressed by appropriate surgical intervention in a timely fashion. RRD patients, even if managed with elegant surgical skill resulting in successful anatomic reattachment, often suffer visual disability with final post-operative visual acuity less than 20/200 [2]. Studies evaluating outcomes in RRD patients suggest a final anatomic success rate (reattachment) in about 95% of cases [3]. Though studies show variability, approximately 50% of patients achieve visual acuity better than 20/50 (reading vision), while the remaining 50% can expect visual acuity less than 20/50 [2]. To the authors’ knowledge, no studies have evaluated visual outcomes in AMD patients with RRD.

AMD is a common degenerative disorder affecting precisely the tissues affected by RRD [4], and thus its presence might portend poorer outcomes for RRD patients. The earliest and most persistent visual decline in AMD is delayed rod-mediated dark adaptation, an indicator of photoreceptor sustenance across the choriocapillaris-Bruch’s membrane-RPE interface [5, 6]. This dysfunction is attributed to the age- and disease-related loss of choriocapillaris endothelium and lipidation and cross-linking of Bruch’s membrane, new layers of extracellular deposits on basal and apical aspect of the RPE, with the latter directly impacting the RPE-photoreceptor interface. Cone-mediated visual acuity, a routine clinical test, may remain good until late in progression, attributed to maintenance by foveal Müller glia and the retinal circulation [7]. AMD could thus impact the visual prognosis for patients following successful RRD repair via several mechanisms, including reduced oxygen and nutrition due to increased pathlength from failing choriocapillaris and disturbed interaction between RPE apical processes and outer segments.

Data regarding expected visual acuity after RRD in patients with AMD would be important both for patients and physicians counselling these patients preoperatively. What can patients with the already potentially visually devastating diagnosis of AMD expect for their vision following RRD repair? How should surgeons manage the preoperative discussion and post-operative recovery expectations of these patient? Herein, we present our visual outcomes in AMD eyes vs. normal eyes.

## Methods

Institutional Review Board (IRB) approval was obtained from the University of Alabama at Birmingham prior to collection of patient data. The study was HIPAA-compliant and adhered to the tenets of the Declaration of Helsinki.

In this retrospective cohort study, we reviewed 1149 patient charts. These patients presented to retina specialists at Retina Consultants of Alabama and had retinal detachment repair at Callahan Eye Hospital at the University of Alabama at Birmingham. Eyes with primary macula-off RRD that underwent surgical repair between years 2013-2017 were included. Patients underwent pars plana vitrectomy (PPV), PPV with scleral buckle, or primary scleral buckling at the discretion of the surgeon. Tamponade agents including sulfur hexafluoride (SF6), perfluoropropane (C3F8), and silicone oil were used at the discretion of the surgeon. Exclusion criteria were as follows: macula-on RRDs, exudative RDs (retinal detachments), tractional RDs, recurrent RDs, eyes with less than 6 months of follow up, or eyes with pre-existing retinal disease (other than AMD). Because AMD patients tend to be older than non-AMD patients, both groups were limited to patients aged 55 or older. A total of 191 eyes met study criteria, of which 162 were non-AMD (controls) and 29 were AMD.

Demographic information, including patient age and lens status, was compared between the two groups (See Table 1). Primary outcome measures included a final Snellen visual acuity categorized as 20/40 or better vision, worse than 20/40 and better than 20/200 vision, and worse than 20/200 vision (See Table 2). Patients with Count Fingers (CF), Hand Motion (HM), Light Perception (LP), and No Light Perception (NLP) vision were analyzed as separate from patients with 20/200 or worse vision because of the categorical nature of these visual acuity measures. Surgical information was also collected as part of the chart review (See Table 3).

The Wilcoxon rank sum test was used to compare continuous variables between those with and without AMD. Categorical comparisons were made between the two groups using Fisher’s exact test. The level of significance was 0.05.

## Results

Demographic information is included in Table 1. The median age of patients with AMD, as expected, was significantly older (*p* < 0.001) than patients without AMD even with the age restriction of older than 55 years. There was a significant difference in lens status between the two groups with those with AMD having higher frequency of pseudophakia (*p* = 0.0379).

**Table 1:** Demographic information of AMD and non-AMD patients that underwent RRD repair

	Non-AMD (***n*** = 162)	AMD (***n*** = 29)	***p***-value
**Age (years)**			
**Mean**	64.8	74.9	**< 0.0001**
**Median**	63.2	75.6	
**Lens status**			**0.0379**
**Phakic**	68 (42.0)	6 (20.1)	
**Pseudophakic**	94 (58.0)	23 (79.3)	

Table 2 shows the preoperative and postoperative visual acuity by AMD status. Preoperative visual acuities did not differ significantly between the AMD and non-AMD groups (*p* = 0.8239), or between neovascular AMD (NVAMD) and dry AMD groups (*p* = 0.7112). Of the 29 eyes in the AMD group, 4 had NVAMD, and 25 had dry AMD. There was a statistically significant difference in postoperative visual acuity by AMD status, with those without AMD having a worse visual outcome overall (*p* = 0.0048). However, those without AMD had a higher frequency of Count Fingers (CF), Hand Motion (HM), Light Perception (LP), or No Light Perception (NLP) vision (*p* = 0.023). More specifically 5.6% of non-AMD eyes and 3.5% of AMD eyes were 20/40 or better, 77.2% of non-AMD and 55.2% of AMD eyes were worse than 20/40 and better than 20/200, 10.5% of non-AMD eyes and 37.9% of AMD eyes were 20/200 or worse, but not CF or worse. As mentioned, there were 11 eyes in the non-AMD group with CF, HM, LP, or NLP vision while there was only 1 eye in the AMD group with CF vision, and this was statistically significant (*p* = 0.023). There was no difference in postoperative visual acuity in the AMD group between AMD subtypes (*p* = 0.6908). The median follow-up time for final visual acuity was 11 months.

**Table 2:** Preoperative and postoperative visual acuity by AMD status

	Non-AMD (***n*** = 162)	AMD (***n*** = 29)	***p***-value
**Pre-operative BCVA, n (%)**			0.8239
**>** **20/40**	0	0	
**< 20/40 to** **>** **20/200**	33 (20.4)	7 (24.1)	
**< 20/200**	36 (22.2)	7 (24.1)	
**CF**^a^**, HM**^b^**, or LP**^c^	93 (57.4)	15 (51.7)	
**Post-operative BCVA, n (%)**			0.0048
**>** **20/40**	9 (5.6)	1 (3.5)	
**< 20/40 to** **>** **20/200**	125 (77.2)	16 (55.2)	
**< 20/200**	17 (10.5)	11 (37.9)	
**CF, HM, or LP**	11 (6.8)	1 (3.5)	

^a^CF: Count Fingers, ^b^HM: Hand Motion, ^c^LP: Light Perception

Surgical information is detailed in Table 3. The final reattachment rate was 93.8% in the non-AMD group and 96.6% in the AMD group. The AMD and non-AMD groups did not differ significantly in the frequency of final reattachment (*p* = 1.000), in surgical approach (PPV, PPV/Buckle, or Primary Buckle) (*p* = 0.7903), in tamponade agent (*p* = 0.4295), or in the frequency of complications, as listed in Table 4.

**Table 3:** Surgical information by AMD status

	Non-AMD	AMD	*p*-value
Final Attachment Rate, n (%)	152 (93.8)	28 (96.6)	1.000
Surgical Approach, n (%)			0.7903
PPV	152 (93.8)	29 (100)	
PPV/Buckle	6 (3.7)	0	
Buckle	4 (2.5)	0	
Tamponade Agent, n (%)			0.4295
SF6	109 (67.3)	23 (79.3)	
C3F8	5 (3.1)	0	
SO	44 (27.2)	5 (17.2)

**Table 4:** Complications by AMD status

Complications n (%)	Non-AMD	AMD	*p*-value
Endophthalmitis	0	0	–
Vitreous Hemorrhage	9 (5.6)	1 (3.5)	1.00
Choroidal Hemorrhage	2 (1.2)	0	1.00
Proliferative Vitreoretinopathy	32 (16.8)	2(1.1)	0.117

## Discussion

RRD is an alarming diagnosis for any patient. Arguably, it may be of even greater concern to the AMD patient already at risk of losing central vision over time. In addition, managing patient expectations in the preoperative counselling session and going forward over the course of postoperative patient visits is a vital part of patient care. The visual future is admittedly a bit unsure for any patient with RRD, but published literature gives the retina surgeon some guidance as to how to best prepare and inform patients undergoing RRD repair.

Several studies reported visual acuity outcomes for macula-off RRD repair in non-AMD patients. In a study of macula-off RD repair with scleral buckling (SB) by Ross and Kozy, 59% of eyes had visual acuity greater than or equal to 20/50, 35% had visual acuity between 20/60 and 20/200, and 5% had visual acuity less than 20/200 with no significance difference in final visual acuity in regards to timing of repair within first week of detachment [2]. Salicone et al. reported that out of 457 total macula-off RRDs repaired by SB, 27.8% of eyes had visual acuity greater than or equal to 20/40, 25.2% had visual acuity between 20/50 and 20/100, and 47% had vision less than or equal to 20/200 [8]. In another study of 164 macula-off RRDs repaired by SB, Ahmadieh et al. found 13.4% of eyes with visual acuity better than or equal to 20/40, 17.7% between 20/50 and 20/100, 40.9% between 20/200 and 20/400, and 28% count fingers or worse [9].

A number of other studies have also evaluated visual outcomes in macula-involving RRD repaired by pars plana vitrectomy (PPV). In such a study of 178 eyes, Campo et al. found that 65% of eyes with the macula detached fewer than 30 days had visual acuity greater than or equal to 20/50 while 41% of eyes with the macula detached greater than 30 days had visual acuity greater than or 20/50 [10]. The mean final visual acuity was 20/40. Mendrinos et al. reported among 44 eyes, 38.6% saw 20/40 or better and 47.7% saw 20/50 or better [11]. Finally, in a study analyzing macula-off RRD repaired by PPV, SB, or PPV combined with SB, Pastor et al. reported on 349 patients with macula-off RRDs and found that 28.4% saw 20/40 or better, 44.2% saw between 20/50 to 20/100, and 27.4% had visual acuity worse than 20/100 [12]. In our study, fewer patients achieved vision better than 20/40 in both the AMD and the control group as compared to the aforementioned studies (5.56% in the control group and 3.45% in the AMD group). However, a higher percentage of patients achieved final vision between 20/40 and 20/200 as compared to prior studies (77.16% in the control group and 55.17% in the AMD group).

Herein, we present to our knowledge the first data on visual acuity outcomes in AMD patients with RRD. There was a statistically significant difference in postoperative visual acuity by AMD status (*p* = 0.0048) with those without AMD having worse vision overall. We attribute this result to the fact that those without AMD had a higher frequency of CF, HM, LP, or NLP vision (*p* = 0.023) and thus worse postoperative visual acuity. The finding that non-AMD eyes had worse overall postoperative acuity is not likely a clinically significant finding, but rather a function of the difference in sample size and composition between the two groups. A higher percentage of patients in the control group had silicone oil tamponade, though this difference was not statistically significant. As silicone oil is often used in more complex retinal detachment repairs, this could suggest a reason as to why more eyes in the control group had worse visual acuity outcomes. Neither group (control or AMD) was exceptionally large due to the need to exclude many eyes with confounding variables. The AMD group was significantly smaller than the control group because of the challenge of finding eyes with both AMD and RRD. Accordingly, a larger sample in the control group would likely have found fewer eyes with severe RRD causing CF, HM, LP, or NLP vision. Similarly, a larger AMD group would likely have found more eyes with severe RRD and poor vision. Rather than suggesting that control eyes did worse, this result implies that AMD eyes had comparable visual outcomes to non-AMD eyes. In fact, though the sample size was small as mentioned, a similar percentage of patients in both groups achieved better than 20/40 vision (5.6% in the non-AMD group, and 3.5% in the AMD group). Importantly, this study suggests AMD patients can expect similar outcomes to non-AMD patients after RD repair. This informs patients and clinicians that the double insult to the retina of AMD and RRD involving the macula does not necessarily mean a poor visual prognosis. Based on our data, approximately 58% of patients will maintain functional vision better than 20/200 after RD repair even in the presence of AMD. Moreover, the vast majority of patients in the AMD group (as well as the non-AMD group) had an improvement in visual acuity after surgery (65.5% of AMD eyes, and 75.9% of non-AMD eyes, *p* = 0.2103).

This information gives surgeons a starting point for the discussion guiding patients’ expectations following surgery. This also gives some hope to these patients, suggesting that there is certainly at least a reasonable chance for visual improvement.

Limitations of our study include its retrospective nature, the small number of patients with both AMD and macula-off RRD, and the lack of vision tests beyond cone-mediated acuity. The inability to age-match the two samples (due to the older age of AMD patients) is also a limitation. The difference in lens status (more pseudophakia in the AMD group) is also a limitation, though this is expected in the older AMD population. Strengths of the study include the presence of a comparison group without AMD and the fact that this study to our knowledge is the first of its kind. Future studies with larger samples sizes will further clarify the visual prognosis for patients with AMD and RRD. In particular, a study to evaluate the correlation of duration of macular detachment with visual outcomes in AMD patients as compared to controls would be instructive. Studies have demonstrated that duration of detachment is correlated with visual outcomes [13], and one could hypothesize that such an affect may be even greater in AMD eyes.

## Conclusions

In conclusion, the diagnosis of concomitant AMD and RRD may not be a devastating diagnosis visually. It appears that the routine use of best surgical practice to manage RRD in AMD is well worth the effort for patients. Though clinicians should be careful not to overestimate visual outcomes, patients can be assured that surgical intervention does offer the hope of functional vision.

## Supplementary Information

**Additional file 1.**

## Data Availability

The datasets used and analyzed for this study are available as a supplementary file linked to this article.

## Abbreviations

CF: Count Fingers
HM: Hand Motion
LP: Light Perception
NLP: No Light Perception
RD: Retinal detachment
RRD: Rhegmatogenous retinal detachment
AMD: Age-related macular degeneration
NVAMD: (Neovascular AMD)
SB: Scleral buckle
PPV: Pars plana vitrectomy
